# Fine-tuning GPT-3 for machine learning electronic and functional properties of organic molecules

**DOI:** 10.1039/d3sc04610a

**Published:** 2023-12-05

**Authors:** Zikai Xie, Xenophon Evangelopoulos, Ömer H. Omar, Alessandro Troisi, Andrew I. Cooper, Linjiang Chen

**Affiliations:** a Leverhulme Research Centre for Functional Materials Design, Materials Innovation Factory and Department of Chemistry, University of Liverpool Liverpool L7 3NY UK aicooper@liverpool.ac.uk; b Department of Chemistry, University of Liverpool Liverpool L69 3BX UK; c School of Chemistry, School of Computer Science, University of Birmingham Birmingham B15 2TT UK l.j.chen@bham.ac.uk

## Abstract

We evaluate the effectiveness of fine-tuning GPT-3 for the prediction of electronic and functional properties of organic molecules. Our findings show that fine-tuned GPT-3 can successfully identify and distinguish between chemically meaningful patterns, and discern subtle differences among them, exhibiting robust predictive performance for the prediction of molecular properties. We focus on assessing the fine-tuned models' resilience to information loss, resulting from the absence of atoms or chemical groups, and to noise that we introduce *via* random alterations in atomic identities. We discuss the challenges and limitations inherent to the use of GPT-3 in molecular machine-learning tasks and suggest potential directions for future research and improvements to address these issues.

## Abstract 2

Fine-tuned GPT-3 shows robust performance for the prediction of electronic and functional properties for organic molecules, with resilience to information loss and noise.

## Graphical abstract

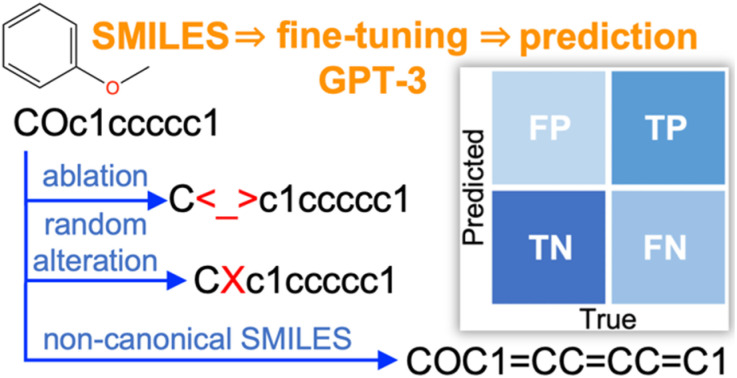

## Introduction

There has been recent and growing interest in leveraging machine learning (ML) for diverse applications involving organic molecules, such as predicting molecular properties^1–6^ and using inverse design techniques to create new functional molecules.^7–10^ Such ML-oriented tasks have facilitated a deeper comprehension of structure–property relationships, led to the discovery of new chemical reactivity, and catalyzed the development of novel functional molecules and materials, including drugs and catalysts.

The advent of the latest large language models (LLMs), notably GPT-3 and GPT-4,^11,12^ has quickly attracted the attention and interest of chemists. Indeed, despite inherent limitations and valid concerns about the way that LLMs operate, GPT models have emerged as tools that offer the potential to transform the way chemists approach their research. LLMs, which are trained on vast amounts of text data, can generate human-like text, answer questions, and even perform tasks that require understanding and reasoning. Used with caution, almost any aspect of chemistry research might benefit from such capabilities, while others may require additional enhancements to the LLMs, such as fine-tuning and the use of plugins.

One of the most significant impacts that LLMs may have on chemistry is their potential ability to accelerate research and discovery by interacting with human chemists. For example, GPT-4 has been integrated into an iterative process of discovering new metal–organic frameworks (MOFs), operating through a cooperative workflow between GPT-4 and a human chemist.^13^ Through structured prompting of GPT-4 and in-text learning informed by human feedback, the human-artificial intelligence (AI) collaboration yielded the discovery of an isoreticular series of MOFs, each synthesized using distinct strategies and optimal conditions.

Sophisticated, LLM-powered AI chemistry agents have been reported to accomplish tasks across organic synthesis, drug discovery, and materials design. One such example is ChemCrow,^14^ a GPT-4-powered chemistry engine designed to streamline the reasoning process for various common chemical tasks, including drug and materials design and synthesis. ChemCrow combines chain-of-thought reasoning with expert-designed tools for chemistry. It operates by sequentially prompting GPT-4 with instructions, guiding it to reason about the current state of the task, consider its relevance to the final goal, and plan the next steps accordingly. ChemCrow has proven to be an effective assistant to expert chemists, while also lowering the entry barrier for non-experts by offering a simple interface to access accurate chemical knowledge.

In addition to their natural language processing and conversational capabilities, extensively pre-trained LLMs have demonstrated significant potential in predicting molecular and material properties, as well as in the inverse design of functional molecules and materials. Task-specific fine-tuning of GPT-3 has resulted in surprisingly effective prediction performances across a range of chemistry ML tasks, often surpassing the performance of dedicated ML models specifically developed for these tasks.^15^ Notably, the fine-tuning of GPT-3 showed exceptional strength in low-data ML tasks. Furthermore, the performance of the fine-tuned GPT-3 models remained robust regardless of the representation used, such as chemical names or line representations like SMILES or SELFIES. This suggests that GPT-3 is adept at extracting correlations from any form of text. However, it is crucial to exercise caution when interpreting the success of such fine-tuned GPT-3 models. Impressive performance likely indicates that the GPT-3 model has identified and exploited correlations in the data for predictions. It does not necessarily imply that these correlations are chemically meaningful or causal.

There is a rapidly growing community of researchers who are exploring ways to leverage LLMs for chemical discovery challenges. A recent thematic hackathon provided 14 compelling examples of how LLMs can revolutionize materials science and chemistry.^16^ These examples spanned a wide array of applications, from predictive modeling to the creation of educational tools, illustrating the models' capacity to go beyond their initial training parameters. The event highlighted the ability of LLMs to extract information from unstructured data and to seamlessly integrate different tools *via* natural language interfaces. The versatility of LLMs displayed in projects for predictive modeling, interface design, knowledge extraction, and educational tool development indicates their potential to enhance workflow efficiency, minimize errors, and increase productivity within scientific fields.

In this study, we hypothesize that the combination of GPT-3's language understanding capabilities and the inherently human-readable nature of the SMILES notation^17^ may enable effective recognition of significant patterns within chemical structures and capture the dependencies of molecular properties on these structures. To test this hypothesis, we approach several molecular property prediction tasks by applying GPT-3 to the classification of SMILES strings. Our aim is to explore GPT-3's ability to discern subtle differences in molecular structures and to accurately classify compounds into specific categories, as defined by their molecular properties.

We focus on assessing the efficacy of fine-tuning GPT-3 for predicting the electronic properties of organic molecules. We use a dataset of organic molecules extracted from the Cambridge Structural Database, previously reported by some of the authors here.^18^ The dataset consists of 48 182 organic molecules, all with documented synthetic pathways and stability in the solid state. Their electronic properties, relevant to semiconductor applications, were determined by quantum chemical calculations. We present results for fine-tuned GPT-3 models in predicting energetics of the frontier molecular orbitals; that is, energies of the Highest Occupied Molecular Orbital (HOMO) and Lowest Unoccupied Molecular Orbital (LUMO). We compare the performance of these GPT-3 models with that of message-passing graph neural networks.^19^ Additionally, we test the robustness of our fine-tuned GPT-3 models against ‘adversarial attacks’, explore the potential explicability of the models in correlating molecular structure with properties, and the evaluate the ability of fine-tuned GPT-3 models to make predictions for ‘unknown’ molecules that were not represented in the training set. Finally, we discuss the limitations and challenges associated with using LLMs in chemical classification tasks and propose potential avenues for future research and improvements.

## Methods

### Background knowledge of GPT-3

Generative Pre-trained Transformer 3 (GPT-3),^11^ developed by OpenAI, is a sophisticated large-scale language generation model. Using a transformer architecture, it employs self-attention mechanisms to manage long-range dependencies within text. The model can generate sentences that harmonize with any given context based on the highest probability. GPT-3 was pre-trained on a wide-ranging corpus of text data, including internet text, books, and articles, under an unsupervised learning framework. This pre-training phase empowered the model to predict the next word in a sentence, thereby facilitating the learning of patterns, structures, and representations in language. With its 175 billion parameters, GPT-3 stands as one of the largest language models currently available. Post pre-training, GPT-3 underwent fine-tuning using task-specific data, preparing it for specific applications such as text generation, machine translation, and question answering. This process optimized the model's capabilities and performance for these specific tasks.

The GPT-3 model incorporates a multi-layered self-attention mechanism, borrowed from the transformer model, into the decoder segment of the encoder–decoder architecture. This allows GPT-3 to capture dependencies among all words in a sentence simultaneously, thus enabling it to comprehend long-range dependencies and contextual information. One of GPT-3's notable features is its ability for zero-shot and few-shot learning. In other words, it can generate coherent text with little or no task-specific training data, indicating its comprehensive understanding of the structure of language. Additionally, GPT-3 exhibits transfer learning, seamlessly applying knowledge from one domain to another. However, as noted frequently by others, GPT-3 may also generate incorrect or nonsensical responses and display biases that are inherent in its training data.

Pre-trained GPT-3 models, such as ‘ada’, can be fine-tuned to specialize in specific tasks or domains using OpenAI's API. Fine-tuning refers to the process of adapting a base model, which has already been pre-trained on a vast corpus of generalized data, to perform better on a more specific task. During this process, the model's parameters are adjusted to minimize errors for the new task. This allows the model to tailor its knowledge for the specific task, enhancing its performance.

### Simplified molecular input line entry system (SMILES)

SMILES is a notation system used in chemistry that provides a compact, human-readable way to represent a molecular structure using ASCII strings.^18^ A SMILES string is composed of atomic symbols and indications of connectivity and is read from left to right. Hydrogen atoms are usually not explicitly represented as it is assumed that they are present as required by the molecule's standard valences.

In a broad sense, SMILES can be considered a type of language, designed to provide a standardized method of writing chemical structures in text form. Like all languages, SMILES has its own syntax (rules about structure) and semantics (rules about meaning). The syntax includes the use of specific characters to represent atoms, bonds, ring structures, and branches, while the semantics define how these characters are interpreted as chemical structures. In this respect, learning to write in SMILES is somewhat akin to learning a new language where understanding the rules and conventions is crucial. However, unlike human languages, the SMILES notation lacks grammar rules that govern word order and does not convey meaning through the arrangement of ‘words’ into ‘sentences’. Each SMILES string represents a single molecule, and in common usage they are not typically read sequentially to extract additional meaning.

### Fine-tuning GPT-3 for molecular ML tasks

When a SMILES string such as ‘c1ccccc1’ for benzene is input into the chat interface of a GPT model, the typical response provided is: “The SMILES string c1ccccc1 represents benzene, a simple aromatic compound with a ring structure.” This response reflects the GPT model's stored knowledge, which does not extend to predicting the electronic or functional properties of molecules. To enable the GPT model to predict such properties, it must be fine-tuned with data that is specific to the task at hand, such as molecular property prediction.

All our fine-tuned GPT-3 models made use of the “ada” base model. The training data points were structured as prompt-completion pairs, following the format given below, and stored in JSONL files:

{"prompt":"SMILES","completion":"property class label"}.

In this format, the SMILES string of a molecule serves as the prompt, which is then completed with a class label assigned to the molecule for a specific property (*e.g.*, its HOMO value). The property class labels were categorized as 0/1, 0/1/2, and 0/1/2/3 for binary, ternary, and quaternary classifications, respectively.

**Fig. 1:**
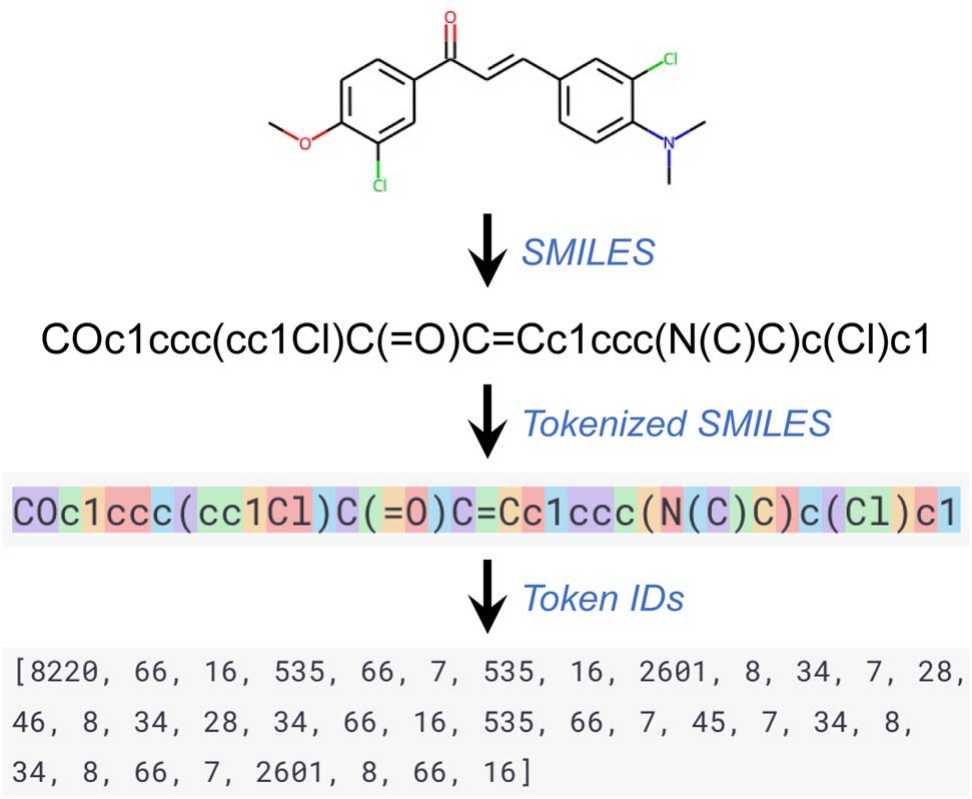
Tokenization of SMILES strings for GPT models.

The GPT series of models, like other language processing models, use a step known as tokenization as part of their preprocessing. In this process, a text segment is divided into smaller units known as tokens, which can range in size from a single character to an entire word. Each token is subsequently converted into a unique ID using a vocabulary list on which the model has been trained. Every word or character within this vocabulary list corresponds to a unique ID. This series of token IDs is then input into the GPT model for processing. The model employs these token IDs to comprehend the structure and semantics of the input text and produce an equivalent output. This output, also a series of token IDs, is ultimately converted back into text (that is, detokenized) for user readability. Throughout this work, the default tokenizer of the GPT API was used. Fig. 1 provides an illustration of a tokenized SMILES string and its corresponding sequence of token IDs.

## Results and discussion

### Machine learning molecular properties

We focused primarily on a dataset of organic semiconductor (OSC) molecules extracted from the Cambridge Structural Database (CSD),^18^ which is referred to as the OSCs dataset hereafter. This dataset comprises 48 182 organic molecules, each accompanied by its SMILES representation and several quantum-chemically computed electronic properties. We fine-tuned the “ada” base model of GPT-3 for multiclass classification tasks on HOMO and LUMO values. Class thresholds were determined by values that equally segmented the property (HOMO or LUMO) value range into the required number of classes. The entire dataset was randomly split into two sets, with 80% of the data allocated for fine-tuning and the remaining 20% reserved for hold-out validation.

All overall accuracy values, reported in Table 1, are for predictions on the hold-out validation set. For the ternary classification of HOMO and LUMO, the fine-tuned GPT-3 models achieved high prediction accuracies of 0.92 and 0.94 respectively. However, as the number of classification classes increased from 3 to 5, the performance of fine-tuned GPT-3 models was noticeably impacted, as indicated by the significantly lower prediction accuracies for HOMO predictions (Table 1). This suggests inherent limitations in the applicability of fine-tuning GPT-3 for molecular ML tasks. For example, such models might be applicable for inexpensively sorting large numbers of candidate molecules into batches for subsequent electronic structure calculations—for example, to identify molecules that are likely to have ‘high’ or a ‘low’ HOMO (or LUMO) energies, or a narrow optical gap (*e.g.*, high HOMO, low LUMO pairs), but such models are unlikely to be useful for near-quantitative predictions, for which a much larger number of classifications classes would be required.

**Table 1:** Fine-tuning GPT-3 for molecular ML tasks

Dataset	Size	Prediction task	Data split^a^	Number of classes	GPT-3 accuracy^b^	GNN accuracy	Descriptors accuracy^c^
OSCs	48 182	HOMO	Train : test = 0.8 : 0.2	3	0.92	0.94	0.87
OSCs	48 182	HOMO	Train : test = 0.8 : 0.2	4	0.68	0.75	0.47
OSCs	48 182	HOMO	Train : test = 0.8 : 0.2	5	0.60	0.68	0.40
OSCs	48 182	LUMO	Train : test = 0.8 : 0.2	3	0.94	0.94	0.91
AMPs	572	HER	Stratified 10-fold	2	0.88	0.86	0.87

a For each of the ML tasks on the OSCs dataset, the same training and test (hold-out validation) data were used by GPT-3 and GNN models.

b GPT-3 was independently fine-tuned for each ML task.

c The RDKit function CalcMolDescriptors() was used to calculate all available descriptors. Molecules for which significantly fewer descriptors were calculated were excluded; descriptors with missing values for any molecule were also discarded. Afterwards, feature selection was done using the SelectKBest (*K* = 20) method in scikit-learn. The resultant features were scaled individually to the range of [0,1]. Finally, an SVM classifier was trained for the specific task.

A graph neural network (GNN) was chosen to be a baseline model for benchmarking the performance of fine-tuned GPT-3 models on the same molecular ML tasks. All GNN-based results reported here were obtained using the Chemprop package, which implements a directed message passing neural network (D-MPNN).^20^ Chemprop's D-MPNN has demonstrated robust capabilities in predicting molecular properties across a range of topics, from computed electronic properties to protein binding affinities and to molecular toxicities. We used the default molecular graph representation generated by Chemprop, without augmenting it with any additional atom-, bond-, or molecule-level features.

Table 1 shows that for ternary classification of HOMO and LUMO on the OSCs dataset, the fine-tuned GPT-3 models performed on par with the trained GNN models. However, GPT-3 slightly underperformed compared to the GNN models on the 4-class and 5-class classification tasks for HOMO. This is perhaps unsurprising, as essentially both the SMILES representation input into GPT-3 and the molecular graph representation input into the GNN encode the same information regarding a molecule's atoms and their connectivity.

**Fig. 2:**
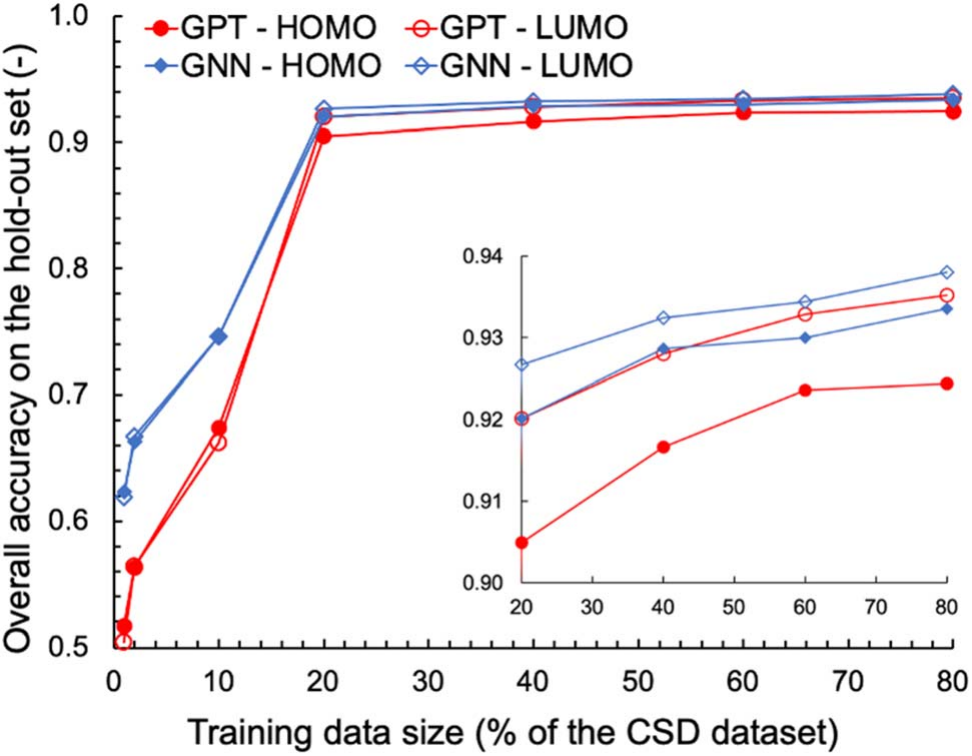
Learning curves for ternary classifications of HOMO and LUMO by fine-tuned GPT-3 and trained GNN models. The inset provides a close-up view of the curves for training data sizes that comprise 20% or more of the complete OSCs dataset.

We also explored the dependence of GPT-3's prediction performance on the size of the data used for fine-tuning. We fine-tuned GPT-3 for ternary classifications of HOMO and LUMO using various fractions of the complete OSCs dataset, ranging from 1% to 80% of the 48 182 data points. For comparison, GNN models were trained on the same classification tasks using the same training data as for the fine-tuning of GPT-3. The learning curves obtained for the various machine learning tasks and models are shown in Fig. 2. With fewer than 1000 training data points (1% and 2% of the OSCs dataset), the fine-tuned GPT-3 models performed poorly, achieving accuracies below 0.6 on the hold-out validation data. However, significant improvements in prediction performance were observed when the size of the training data increased to 20% of the complete OSCs dataset, with prediction accuracies exceeding 0.9 for both HOMO and LUMO classifications. Further expanding the training data size up to 80% of the OSCs dataset only marginally improved the prediction performance of the fine-tuned GPT-3 models, achieving accuracies of 0.92 and 0.94 for HOMO and LUMO, respectively. The GNN's prediction performance was nearly equivalent to that of GPT-3 when the training data size was 20% or larger. However, GNN outperformed GPT-3 in the low-data region. This may in part be attributed to two factors: (1) the molecular graph representation being chemically more expressive than SMILES for the ML tasks, and/or (2) the fine-tuning of GPT-3 necessitating a sufficient amount of data to capture the patterns in SMILES relevant to the ML tasks.

In addition to the results from the OSCs dataset, Table 1 also presents results from both fine-tuned GPT-3 models and GNN models for a second molecular dataset. This dataset includes 572 aromatic organic molecules that were experimentally assessed by some of the authors here for sacrificial photocatalytic hydrogen evolution.^21^ Employing the same procedures and setups as for the OSCs dataset, we fine-tuned GPT-3 models and trained GNN models to predict hydrogen evolution rates (HERs) for these aromatic molecular photocatalysts (AMPs). To compare the performances of these GPT-3 and GNN models with the ML studies from the original work, we implemented stratified 10-fold cross-validation. The fine-tuned GPT-3 achieved an accuracy score of 0.88, slightly outperforming the GNN's score of 0.86 and closely matching the highest prediction performance (0.89 accuracy) reported in the original work. It is interesting that GPT-3 is competitive here with ML models in the original work^21^ that made use of engineered, domain-specific features to encode molecular electronic properties.

Another baseline was established to benchmark the performance of the fine-tuned GPT-3 on the molecular ML tasks, as shown in Table 1. This involved calculating all available molecular descriptors for the molecules in the dataset (either OSCs or AMPs) from SMILES using the RDKit package, which yielded over 200 descriptors for most molecules. Molecules for which significantly fewer descriptors were calculated were excluded. Similarly, descriptors with missing values for any molecule were also discarded. The resulting complete matrix was subjected to a feature selection process using the SelectKBest method within the scikit-learn package, retaining the top 20 descriptors as determined by univariate statistical tests. A support vector machine (SVM) classifier was then trained for each specified ML task. Table 1 shows that the descriptor-based models consistently underperformed in the molecular ML tasks compared to the fine-tuned GPT-3 and GNN models.

These results indicate that fine-tuning GPT-3 could be an effective approach for ML tasks related to molecular properties. It may be particularly advantageous given that fine-tuning GPT-3 requires minimal effort in preparing ML inputs, compared to the effort required in designing and calculating molecular features or, to a lesser extent, generating molecular graphs. However, GPT-3 functions as a “black box” with only a few parameters available for adjustment, and as such it does not provide physical insight or explainability like ML models trained on engineered, physicochemical features. Nor does it offer the same level of explicability that is possible with GNN models.

### Ablation study 1: single-atom removal

We next conducted a series of ablation tests, where certain sections of the SMILES prompts were systematically removed or ‘ablated’ to assess the robustness of the fine-tuned GPT-3 models against information loss. By comparing the predictions using the complete prompt (*i.e.*, complete SMILES strings) to those of the ablated versions (with certain parts of the SMILES strings removed), we aimed to (i) determine if the fine-tuned GPT-3 models had learned chemically meaningful patterns, rather than merely “memorizing” the training data, and (ii) get a sense of the inner workings of the models.

**Fig. 3:**
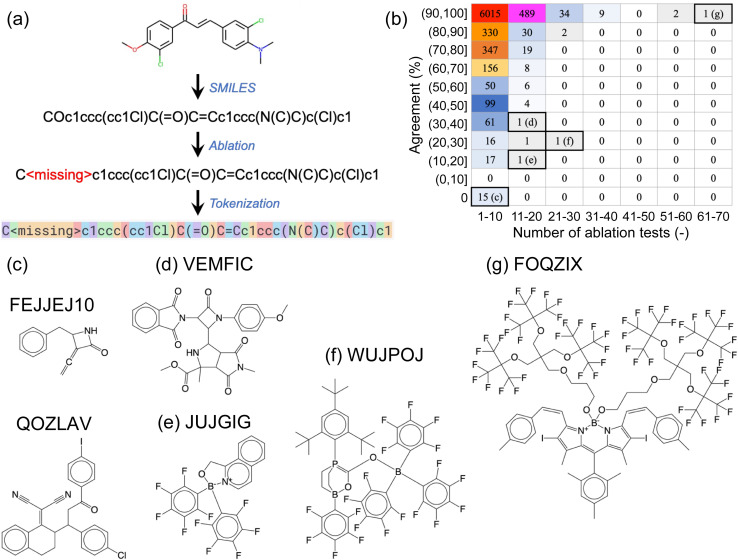
(a) Illustration of single-atom ablation tests, where one non-hydrogen, non-carbon atom is removed from the SMILES string and replaced with a designated empty token, <missing>. (b) A breakdown of results for the 45 763 ablation tests conducted on 7714 SMILES strings. The horizontal axis indicates the number of ablation tests conducted on a specific molecule, and the vertical axis represents the agreement rate between predictions based on complete and ablated SMILES strings. For instance, if a molecule contained three non-hydrogen, non-carbon atoms to be ablated and one out of the three ablated SMILES strings yielded the same prediction as the complete SMILES string (*i.e.*, a 33% agreement rate), this molecule would be counted towards the table element that corresponds to 1–10 ablation tests and an agreement rate in the range of (30%, 40%]. The numbers displayed within the table represent the numbers of molecules categorized by the respective table elements. (c)–(g) Representative molecules corresponding to the labeled table elements in (b).

The first type of ablation test involved single-atom removal: each of the non-hydrogen (H), non-carbon (C) atoms in a SMILES string was removed, one at a time, and these ablated SMILES strings were used as prompts for the corresponding fine-tuned GPT-3 model (Fig. 3a). For the example SMILES shown in Fig. 3a, five ablated SMILES strings would be created, each with either one of the two oxygen atoms, one of the two chlorine atoms, or the nitrogen atom removed. We used a designated empty token, denoted as <missing>, to replace the atom being ablated. The non-hydrogen, non-carbon atoms involved in the complete OSCs dataset included elements: boron (B), nitrogen (N), oxygen (O), fluorine (F), silicon (Si), phosphorus (P), sulfur (S), chlorine (Cl), arsenic (As), selenium (Se), bromine (Br), and iodine (I).

All ablation tests were conducted using the fine-tuned GPT-3 model for ternary classification of HOMO, which was trained using 80% of the complete OSCs dataset. These ablation tests were performed on all data points in the 20% hold-out validation set that were correctly predicted using complete SMILES strings. As a result, 7714 SMILES strings were examined, leading to a total of 45 763 single-atom-removal ablation tests. Out of these 45 763 ablated SMILES strings, 43 588 tests (95.2%) yielded the same classification predictions as their corresponding complete SMILES strings. This finding suggests that the fine-tuned GPT-3 model was resilient to minor information loss in the text prompts, indicating a degree of robustness.

Fig. 3b provides a breakdown of the 45 763 ablation tests conducted on the 7714 SMILES strings. The vast majority (7106) of these SMILES strings underwent no more than 10 ablation tests each (as shown in the first column of the table in Fig. 3b), meaning that they contained no more than 10 non-hydrogen, non-carbon atoms. Out of these, 6015 SMILES strings remained unaffected by the removal of a single atom, as the agreement between predictions based on complete and ablated SMILES strings was 100% for all of them (these SMILES strings contained 1 to 10 non-hydrogen, non-carbon atoms). Conversely, 15 SMILES strings, each containing between 1 and 5 non-hydrogen, non-carbon atoms, were found to be highly sensitive to single-atom ablations, yielding a 0% agreement rate. Two of these molecules are shown in Fig. 3c, with their corresponding CSD reference codes labeled.

As SMILES strings contain increasingly more non-hydrogen, non-carbon atoms, they generally become less problematic for the fine-tuned GPT-3 model to predict correctly, with a few exceptions such as the molecules shown in Fig. 3d–f. Our visual inspections of the molecules with 11 to 20 atoms to ablate suggests that the high number of ablatable atoms relative to the size of the molecule of different elemental types makes it challenging for GPT-3 to handle. However, this empirical ‘rule’ does not always hold, as demonstrated by the molecule in Fig. 3g, which has the largest number of atoms (69 atoms of 5 elemental types) to ablate in this set, yet it yielded 100% agreement between complete and ablated SMILES strings.

### Ablation study 2: single-group removal

**Fig. 4:**
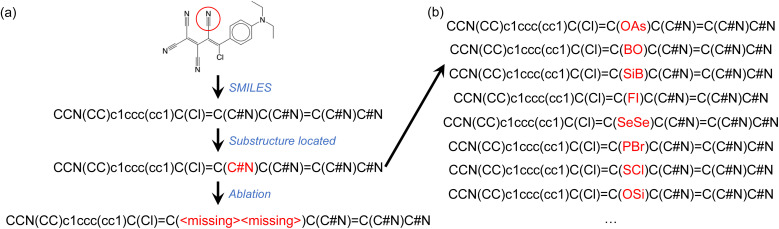
(a) Illustration of single-group ablation tests, where a chemical group is identified (in this case a nitrile group) using its SMARTS notation and replaced with <missing> annotations for atoms belonging to the chemical group. (b) Instead of ablating atoms from the SMILES string, each atom belonging to the target chemical group was replaced with an atom of a randomly selected element type (B, N, O, F, Si, P, S, Cl, As, Se, Br, or I). For each of several investigated molecules, 100 such random variants of the SMILES string were tested.

The second type of ablation test we conducted involved the removal of specific chemical groups from SMILES strings such as, for example, a nitrile group. This was done by replacing the atoms involved in the chemical group with <missing> annotations, as illustrated in Fig. 4a. We considered 15 different chemical groups, which are listed in Table 2. To locate and identify substructures representing the intended chemical groups in the complete SMILES strings, we used the SMARTS representation and the RDKit package. To ensure a sufficient number of molecules containing each chemical group for ablation tests, we fine-tuned the GPT-3 models using 40% of the complete OSCs dataset, reserving the remaining 60% of the data for ablation tests. As shown in Fig. 2 and discussed above, GPT-3 models fine-tuned with 40% of the complete OSCs dataset achieved comparable predictive abilities to those fine-tuned with 80% of the dataset. We fine-tuned GPT-3 for ternary classifications of both HOMO and LUMO values.

**Table 2:** Single-group ablation tests

Chemical group	SMARTS	HOMO		LUMO	
		No. of tests	Agreement (%)	No. of tests	Agreement (%)
Nitrile	[NX1]#[CX2]	1833	91	1831	94
Nitro	[$([NX3](=O)=O),$([NX3+](=O)[O-])][!#8]	3485	86	3968	93
Imine	[$([CX3]([#6])[#6]),$([CX3H][#6])]=[$([NX2][#6]),$([NX2H])]	1780	97	1867	97
Enamine	[NX3][$(C=C),$(cc)]	16 747	85	16 817	92
Ketone	[#6][CX3](=O)[#6]	4647	87	5015	96
Carbonyl with nitrogen	[OX1]=CN	4234	91	4521	97
Carbonyl with oxygen	[CX3](=[OX1])O	4940	93	5260	96
Thiol	*-[S;D1]	57	91	56	79
Thiocarbonyl	*=[S;D1]	1452	92	1455	94
Sulfone	[$([#16X4](=[OX1])(=[OX1])([#6])[#6]),$([#16X4+2]([OX1-])([OX1-])([#6])[#6])]	236	90	262	91
Sulfonic acid	*-[S;D4](=O)(=O)-[O;D1]	39	97	28	82
Sulfonate	[$([#16X4](=[OX1])(=[OX1])([#6])[OX2H0]),$([#16X4+2]([OX1-])([OX1-])([#6])[OX2H0])]	69	97	95	95
Sulfonamide	[$([#16X4]([NX3])(=[OX1])(=[OX1])[#6]),$([#16X4+2]([NX3])([OX1-])([OX1-])[#6])]	351	89	383	97
Acetylene	*-[C;D2]#[C;D1;H]	83	81	77	96
Halogens: F, Cl, Br, I	*-[#9,#17,#35,#53]	7131	90	7456	95

Table 2 summarizes the results of the ablation tests performed on the 15 chemical groups. Like the single-atom ablation tests, these tests were only conducted on molecules in the hold-out validation set that were correctly predicted by the fine-tuned GPT-3 models. In each test, only one type of chemical group was ablated at a time. If a molecule contained multiple instances of the same chemical group, each was ablated one at a time, leading to a corresponding number of ablation tests. For example, 1833 ablation tests were performed on nitrile-group-containing molecules from the hold-out 60% of the OSCs dataset (that were correctly predicted based on complete SMILES strings). This number exceeds the actual number of these molecules, as some contained multiple nitrile groups. In 91% of these 1833 tests, the HOMO predictions based on SMILES strings with one nitrile group ablated agreed with the predictions using the complete SMILES strings.

Our results suggest that, across the 15 chemical groups probed, the fine-tuned GPT-3 model attributed significant importance to the acetylene, enamine, nitro, ketone, and sulfonamide groups in its HOMO predictions. This is evident as the model altered its HOMO class assignments in more than 10% of the ablation tests for each of these groups. For LUMO predictions, the fine-tuned GPT-3 model only altered its LUMO class assignments in more than 10% of the ablation tests for the thiol and sulfonic acid groups. However, the quantities of ablation tests for these two chemical groups were low (56 and 28 respectively), implying that the low agreement rates could be due to the small sample sizes of the tests. One possible interpretation here is that the more ‘important’ functionalities tend to be those that participate in electronic π-conjugation.

We further examined a few molecules by implementing a different test. Instead of ablating the atoms belonging to the chemical group of interest, we replaced them with atoms of randomly selected elemental types (Fig. 4b). For the molecule shown in Fig. 4, the fine-tuned GPT-3 model correctly assigned the HOMO class to the ablated SMILES string (Fig. 4a). We then generated 100 randomly mutated SMILES strings as shown in Fig. 4b. In 80% of these mutated SMILES strings, the same fine-tuned GPT-3 model failed to assign the correct HOMO class. This observation, which is not unique to the example provided, seems to suggest that the GPT-3 model filled in the ‘missing’ tokens before making the property prediction. This might partially explain the high agreement rates between predictions based on complete and ablated SMILES strings (Table 2). However, there were numerous cases where the fine-tuned GPT-3 model gave identical predictions irrespective of the mutations to the SMILES string.

### Predicting molecular properties for ‘unknown’ molecules

**Fig. 5:**
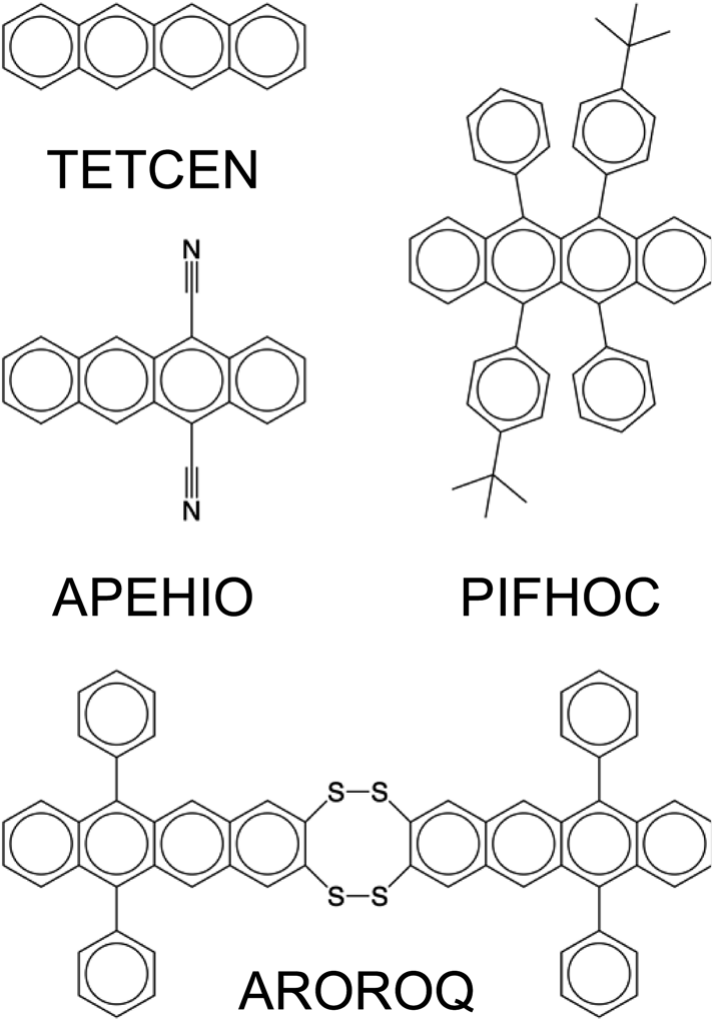
Examples of tetracene-containing molecules in the OSCs dataset, with their CSD reference codes labeled.

We further evaluated the effectiveness of fine-tuning GPT-3 for machine learning molecular properties by generating predictions for unknown molecules. To do this, we identified molecules belonging to families as identified by the presence of common moieties using conjugated molecular fragments. For example, within the OSCs dataset, we found 72 molecules that contained at least one tetracene fragment, as illustrated in Fig. 5. Once we identified such a family of molecules, we excluded all members of that family from both the fine-tuning of a GPT-3 model and the training of a GNN model, hence making this class of molecules effectively ‘unknown’. The remainder of the dataset was then used to train these models. We then used these ML models to predict the target molecular properties for the unknown family of molecules.

Each of the first five families of polycyclic aromatic hydrocarbon-containing molecules (labelled 1–5 in Table 3) was effectively classified by the GPT-3 models, which were fine-tuned without these specific families of molecules. The fine-tuned GPT-3 models demonstrated notably better performance in predicting HOMO than LUMO. Even when all five families of molecules were excluded from the training process, the subsequently fine-tuned GPT-3 models still demonstrated robust performance in predicting their HOMO and LUMO classes. The efficacy of fine-tuning GPT-3 was slightly reduced when predicting ‘unknown’ molecules containing quinones (6–8) or imides (9–11). In all cases, GNN models outperformed their corresponding fine-tuned GPT-3 models marginally.

**Table 3:** Ternary classification accuracies of fine-tuned GPT-3 and trained GNN models for “unknown” molecules

Conjugated fragment	Number of molecules	HOMO accuracy		LUMO accuracy	
		GPT-3	GNN	GPT-3	GNN
Naphthalene (1)	475	0.94	0.95	0.88	0.91
Anthracene (2)	577	0.99	1.00	0.93	0.97
Tetracene (3)	72	0.96	1.00	0.90	0.99
Pyrene (4)	237	0.98	1.00	0.97	0.99
Perylene (5)	41	0.98	1.00	0.98	0.95
(1) + (2) + (3) + (4) + (5)^a^	1402	0.97	0.98	0.93	0.95
*p*-Benzoquinone (6)	295	0.83	0.91	0.87	0.87
1,4-Naphthoquinone (7)	282	0.82	0.91	0.94	0.96
9,10-Anthraquinone (8)	186	0.86	0.91	0.99	1.00
(1) + (2) + (3) + (4) + (5) + (6) + (7) + (8)^b^	2165	0.88	0.91	0.95	0.96
1,8-Naphthalimide (9)	85	0.86	0.93	1.00	1.00
Naphthalenetetracarboxylic diimide (10)	88	0.86	0.88	0.95	0.98
Perylenetetracarboxylic diimide (11)	76	0.85	0.89	0.97	0.97
(1) + (2) + (3) + (4) + (5) + (6) + (7) + (8) + (9) + (10) + (11)^c^	3177	0.88	0.88	0.97	0.97

a All five families of molecules were excluded from model training. The HOMO/LUMO prediction accuracies reported in this row were measured on these five families of molecules.

b All eight families of molecules were excluded from model training. The HOMO/LUMO prediction accuracies reported in this row were measured on the families 6–8 of molecules.

c All 11 families of molecules were excluded from model training. The HOMO/LUMO prediction accuracies reported in this row were measured on the families 9–11 of molecules.

To further test the effectiveness of fine-tuning GPT-3, we excluded families 1–8 of molecules during fine-tuning when predicting for quinone molecules belonging to families 6–8. Similarly, for the imide molecules belonging to families 9–11, we fine-tuned GPT-3 models while excluding families 1–11. Despite further limiting the model's exposure to patterns shared between target molecules and similar ones, the fine-tuned GPT-3 models performed robustly in predicting the properties of the unknown molecules. These more stringent tests further reinforce that fine-tuning GPT-3 can be an effective strategy for ML tasks involving molecular properties.

### Fine-tuning GPT-3 with both canonical and non-canonical SMILES

The results discussed so far have been derived from GPT-3 models fine-tuned on canonical SMILES strings as generated by RDKit. However, multiple valid SMILES strings can represent a single molecule: for example, CCO, OCC, and C(O)C all represent the same ethanol molecule. To address this, canonicalization algorithms are employed to ensure consistency, generating a singular SMILES string for a given molecule from the multitude of possibilities. This unique output, albeit dependent on the specific canonicalization algorithm applied, is referred to as the canonical SMILES.

To assess the performance of GPT-3 that was fine-tuned on canonical SMILES strings against models fine-tuned with non-canonical ones, we employed a GPT-3 model fine-tuned for ternary classification of HOMO. This model, which used an 80 : 20 train-to-test data split for the OSCs dataset, is referenced as the first model in Table 1. For every molecule in the test set (20% of the OSCs dataset, equating to 8578 molecules), we generated 10 valid non-canonical SMILES strings.^22^ The fine-tuned GPT-3 ternary classifier was then applied to predict the HOMO class labels for all 10 SMILES strings of each molecule. This process was designed to evaluate the consistency of HOMO predictions between the canonical SMILES string and the 10 non-canonical versions for each molecule.

**Fig. 6:**
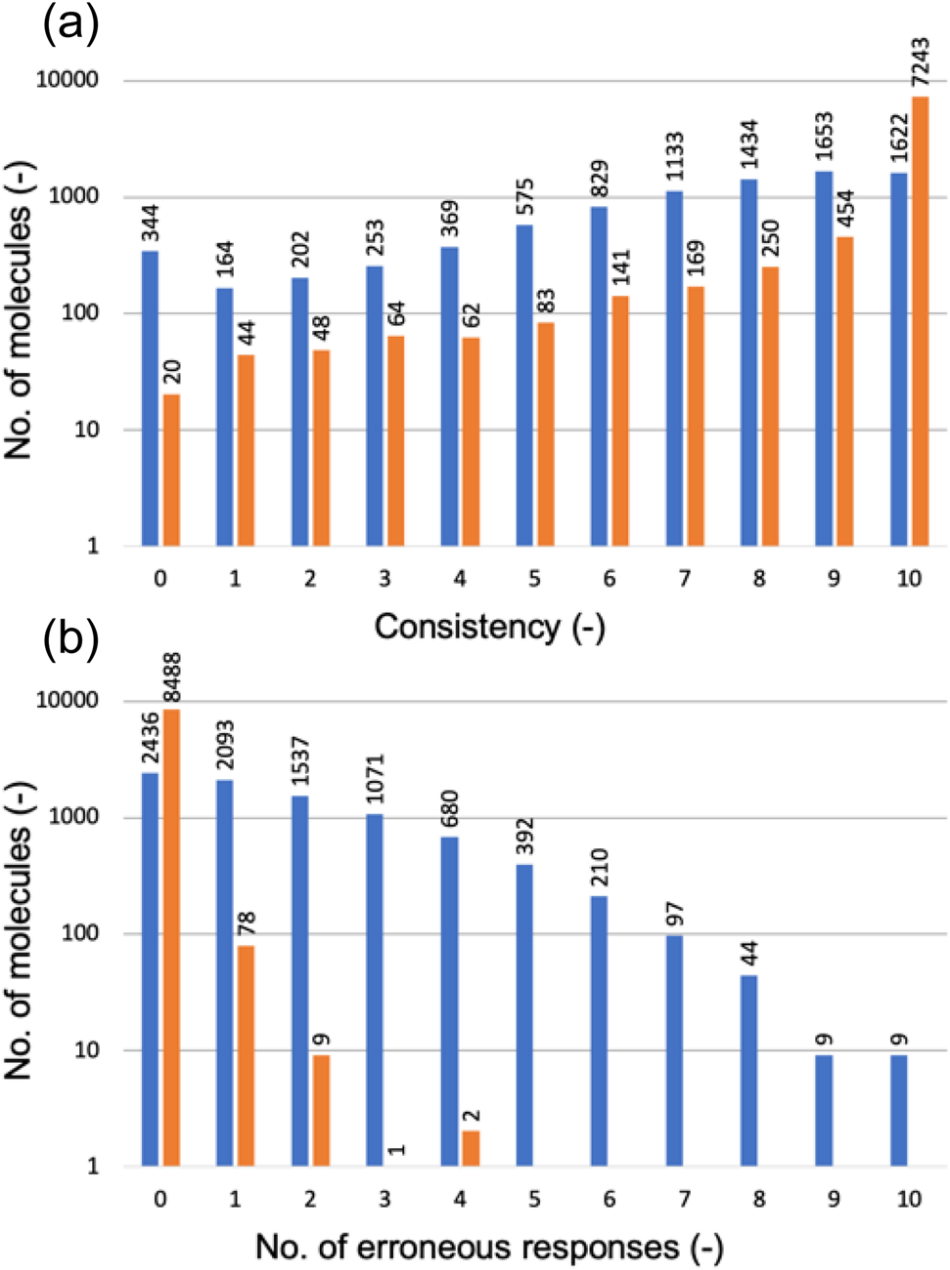
(a) Consistency of HOMO predictions between the canonical SMILES string and 10 non-canonical versions for each molecule in the test set; the consistency level is determined by the number of non-canonical strings receiving the same HOMO class label as the canonical string. (b) Number of erroneous responses to the non-canonical SMILES strings of a molecule; erroneous responses are completions that are not a HOMO class label (0, 1, or 2). In both (a) and (b), the blue bars represent the GPT-3 model fine-tuned exclusively with canonical SMILES strings, while the orange bars represent the GPT-3 model fine-tuned on a dataset augmented with non-canonical SMILES strings. Both vertical axes are in the logarithmic scale.

Fig. 6a categorizes the molecules according to the consistency level, determined by the number of non-canonical strings receiving the same HOMO class label as the canonical string. A consistency level of 0 means that none of the non-canonical strings matched the canonical string's prediction; a level of 10 indicates a perfect match for all. Disappointingly, the GPT-3 model trained solely on canonical SMILES strings performed poorly with non-canonical strings (as shown by the blue bars): for just 1622 out of 8578 molecules, the model predicted consistent class labels across all 11 SMILES variations; for 344 molecules, there was a complete lack of prediction consistency across the SMILES variations.

Furthermore, Fig. 6b (blue bars) reveals that the GPT-3 model often provided erroneous responses to non-canonical SMILES strings, meaning that it completed the prompt with a character that was not a HOMO class label (0, 1, or 2), as it had been fine-tuned to do. Only for 2436 out of 8578 molecules did the model respond with a class label, regardless of whether these were consistent with the canonical reference. For the remainder, the model's completions were incorrect to varying degrees.

The underwhelming performance of the model fine-tuned solely with canonical SMILES strings on non-canonical strings is attributable to the absence of certain patterns in canonicalized SMILES—namely, the different valid permutations of arranging the same group of atoms, as illustrated by the ethanol example above. To address this issue, we expanded the training dataset, which initially comprised only canonical strings, to include five valid non-canonical versions for each molecule. Subsequently, we fine-tuned another GPT-3 model using this augmented dataset and evaluated its performance on the same test set that included each molecule's canonical SMILES string along with 10 non-canonical variants; we followed the same evaluation methodology as used for the model trained only on canonical SMILES strings (represented by the blue bars in Fig. 6).

Fig. 6a demonstrates significant improvements achieved by incorporating non-canonical SMILES strings into the fine-tuning process of GPT-3. For 7243 of the 8578 molecules in the test set, the new GPT-3 model consistently predicted the same HOMO class label across all 11 SMILES versions of each molecule. Across all other consistency levels, this enhanced GPT-3 model—trained with a mix of canonical and non-canonical SMILES—uniformly surpassed the model trained solely on canonical SMILES. Likewise, Fig. 6b shows that the new model produced no erroneous responses for 8488 of the 8578 molecules and very few erroneous responses for the remainder. These results suggest that the enhanced GPT-3 model has benefited from exposure to diverse representations of molecules possible within SMILES notation. It appears that increasing the variety in the training data aids in the fine-tuning process, allowing GPT-3 to develop a more robust understanding of molecular structures and their properties. This makes the model less sensitive to variations in SMILES representation and enhances its ability to generalize from learned patterns. Equally, these results highlight the paramount importance of training data in determining the performance of a fine-tuned model when deployed for predictions.

## Conclusions

Our results suggest that fine-tuning GPT-3, and perhaps other LLMs, can be an effective ML approach to predicting electronic and functional properties of organic molecules, at least in terms of relatively coarse-grained classification tasks. In all ML tasks that we conducted, the fine-tuned GPT-3 model yielded accurate predictions for the hold-out data and even for ‘unknown’ classes of molecules. Moreover, our ablation tests demonstrated the models' resilience against loss of information (due to missing atoms and chemical groups) and noise (random changes in atomic identities). Similar observations of GPT models' robustness and their resilience against noise have also been made by others.^15^ These results lead us to assert that the extensively pre-trained GPT-3, when properly fine-tuned, can detect and distinguish chemically meaningful patterns and discern subtle differences among them, thus effectively ‘specializing’ in the chemistry problems at hand.

This approach has several potential advantages: for example, employing SMILES strings as direct prompts to GPT-3 requires significantly less computational memory compared to many alternative ML input data forms, such as molecular graphs or numerical representations like the Smooth Overlap of Atomic Positions (SOAP) descriptors. Consequently, the GPT-3 fine-tuning approach could be especially advantageous for large molecular datasets composed of millions or even tens of millions of data points.

However, while our findings underscore the potential utility of GPT-3 fine-tuning for predicting molecular properties, there are also certain inherent limitations. First, it does not seem obvious how one might enhance the performance of a fine-tuned GPT-3 model beyond augmenting the training data with more volume and/or diversity, which may not be available for many research goals; indeed, the success of our method here relied on the existence of the large, pre-computed OSC dataset.^18^ This limitation stems from GPT-3's ‘black box’ nature. By contrast, with a molecular graph-based approach, like the directed message-passing neural network used as the baseline in this work, additional chemical information may be incorporated into the graph representation to potentially enhance prediction performance.

Reflecting on the generalized tokenization applied to SMILES in our work (Fig. 1), we hypothesize that a specialized tokenizer that creates chemically relevant tokens—while respecting the chemical nature of the molecular structure and its fragments—could enhance performance in data efficiency and/or prediction accuracy. In a related note, the SELFIES (SELF-referencing Embedded Strings) representation, which often outperforms SMILES in ML tasks, did not show improved performance in our initial tests. This is likely because the generic tokenization applied to SELFIES diminished the extra chemical information that it conveyed compared to SMILES.

Second, while task-specific fine-tuning enables GPT-3 to recognize chemically relevant patterns, thus enhancing its predictive performance, the model does not inherently grasp the chemical principles underpinning the molecular properties. Its predictions are entirely based on pattern recognition and do not imply a deep understanding of the underlying science. This predicament is further complicated because GPT-3 was not yet open-sourced at the time of these studies, which restricts any systematic interpretation of why specific predictions were made. This limits the applicability of this method in scenarios where understanding the reasoning behind a prediction is important. While our ablation tests did shed some light on the importance of certain chemical groups, the findings could be swayed by the underlying assumptions and certainly do not provide a thorough comprehension of the model's decision-making process: in no sense does GPT ‘know’, for example, that a ketone group is prone to conjugation. These challenges relate to model interpretability and, again, greater understanding might be possible to be addressed if a fully open-sourced GPT-3 model were available or if a different, open-sourced LLM was used.

Lastly, fine-tuning LLMs such as GPT-3 can demand considerable resources, making the process both computationally intensive and financially burdensome, particularly for large datasets. In this work, all the fine-tuning tasks conducted *via* OpenAI's API resulted in a total cost of approximately 500 US dollars, excluding the initial exploratory exercises. With other major offerings of LLMs, either fine-tuning is not available, or a local GPU capacity is required for the fine-tuning process (or even pre-training prior to fine-tuning) when applied to chemistry tasks. For now, these hurdles may impede broader testing and/or adoption of LLMs within the chemistry field, following the initial surge of efforts that has centered primarily on GPT models.

In summary, our exploration of fine-tuning GPT-3 demonstrates a promising new approach for predicting molecular properties and, more widely, for discerning patterns in large chemistry datasets. While this strategy has distinct limitations exist, future work in advancing tokenization techniques, improving model interpretability, and reducing computational demands could see LLMs such as GPT-3 becoming an integral part of the chemist's toolkit to complement more traditional computational predictions.

## Data availability

The authors declare that data supporting the findings of this study are available within the paper. All python codes and datasets used in this study are available at https://github.com/XieZikai/Chem-GPT-Finetune.

## Author contributions

L. C. and A. I. C. conceived and supervised the project. Z. X. carried out all the computational work, with support from L. C. and X. E. Ö. H. O and A. T. provided the OSCs dataset and its subsets. L. C., Z. X., and A. I. C. led the preparation of the manuscript.

## Conflicts of interest

There are no conflicts to declare.

## Supplementary Material
